# The good, the bad, and the ugly: a comprehensive study of temperament and personality traits as correlates of self-reported disruptive behavior problems in male and female adolescents

**DOI:** 10.3389/frcha.2023.1173272

**Published:** 2023-05-22

**Authors:** Peter Muris, Ireen Bakker, Myrthe Peulen, Sanne van Mulekom, Cor Meesters

**Affiliations:** Department of Clinical Psychological Science, Maastricht University, Maastricht, Netherlands

**Keywords:** temperament and personality traits, disruptive behavior problems, ODD and CD, HEXACO, impulsivity, effortful control, dark triad

## Abstract

**Purpose:**

The aim of the present study was to provide a comprehensive picture of temperament and personality traits as correlates of self-reported disruptive behavior problems in male and female adolescents.

**Methods:**

Two-hundred-and-sixty-three non-clinical adolescents aged 12–18 years completed a survey containing standardized scales to measure the HEXACO personality traits, impulsivity, effortful control, Dark Triad traits, and symptoms of oppositional-defiant disorder (ODD) and conduct disorder (CD).

**Results:**

The results showed that good traits (in particular, honesty-humility, agreeableness, and the regulative trait of effortful control) were negatively associated, while bad and ugly traits (especially impulsivity and the Dark Triad traits of psychopathy and Machiavellianism) were positively associated with symptoms of ODD and CD. In addition, regression analyses indicated that both types of disruptive behavior problems were associated with a unique set of temperament and personality correlates, and that specific correlates also differed for both genders.

**Conclusions:**

It is concluded that research on the role of temperament and personality should adopt a broad perspective, taking good, bad, and ugly traits from various theoretical models as well as gender differences into account.

## Introduction

Disruptive behavior disorders such as oppositional-defiant disorder (ODD) and conduct disorder (CD) are among the more prevalent psychiatric problems in children and adolescents. A review by Maughan and colleagues (1) has indicated that the prevalence rates in the general population range between 1.0% and 13.3% for ODD and between 0.6% and 13.2% for CD, with considerable variation being noted due to the gender (i.e., males more often display these disorders than females) and age (i.e., ODD mostly has an onset during the early and middle childhood years, while the prevalence of CD shows a clear increase during adolescence), and the used research method (self-report vs. other informant) (2). Both ODD and CD are considered as disruptive behavior problems as the actions displayed by the young person typically cause trouble for the (social) environment and interfere with the usual course of circumstances. In ODD, the child or adolescent exhibits a consistent pattern of angry and irritable mood, argumentative and defiant behavior, and vindictiveness, whereas in CD the behavior is more of an antisocial nature in the sense that the basic rights of others or main societal norms or rules are violated (e.g., by aggressive acts, destruction of property, deceitfulness or theft) (3).

Like other types of psychopathology, the disruptive behavior disorders of ODD and CD are thought to have a multifactorial origin. First of all, it has been demonstrated that genetic factors make an important contribution to ODD and CD problems. For example, in a Swedish nation-wide twin study (*N* = 17,220 twins), it was observed that the correlations of ODD and CD symptoms in monozygotic twins were about twice as large as those found in dizygotic twins, which was translated in heritability coefficients of up to 62% (4). It can be assumed that the genetic vulnerability for these disruptive behavior problems is anchored in the aberrant functioning of specific parts in the brain that are involved in the experience of negative emotion (e.g., amygdala), the processing of punishment and reward (e.g., orbito- and prefrontal cortex), and cognitive control (frontal and parietal cortex) (5). Furthermore, environmental adversities are also thought to increase the risk for ODD and CD, and these include detrimental parenting, other negative familial impact (e.g., abuse, neglect, and violence), socioeconomic hardship, and affiliation with deviant peers (6).

Temperament and personality are also implicated in the etiology of disruptive behavior disorders (7, 8). Both psychological concepts refer to people's habitual ways of behaving and responding across a variety of situations: temperament pertains to the biological make-up of a person and constitutes of individual differences in reactivity (which refers to the excitability and responsivity of the behavioral and physiological systems) and self-regulation (which pertains to neural and behavioral processes that serve to modulate reactivity), whereas personality is concerned with consistent patterns in behavioral, emotional, and cognitive tendencies that arise from temperament but are further formed in interaction with the environment (9). Despite the conceptual difference between temperament and personality, many scholars have pointed out that there are many similarities between both constructs and that many temperament and personality models essentially reflect a similar set of traits (10).

Within the domain of ODD and CD, a variety of temperament and personality traits seem to be relevant. In a recent study, Muris et al. (11) explored the relative contributions of the Dark Triad (12) and the six basic traits of the HEXACO model (13) to disruptive behavior problems in non-clinical adolescents aged 12–18 years (*N* = 117). The Dark Triad consists of three traits reflecting the malevolent nature of human beings, namely psychopathy (which is characterized by persistent antisociality, diminished empathy and remorse, and disinhibited and bold behavior), Machiavellianism (which reflects a deceitful, manipulative interpersonal style with a focus on self-interest and personal gain), and narcissism (which refers to excessive self-involvement, self-aggrandizement, and an extreme focus on the fulfilment of one's personal needs), whereas the HEXACO essentially incorporates the more common temperament/personality traits that are also known as the Big Five (i.e., emotionality, extraversion, agreeableness, conscientiousness, and openness to experience) (14) supplemented with the factor of honesty-humility (which is characterized by fairness, modesty, sincerity, and lack of greed). The results of the Muris et al. (11) study showed that in particular the Dark Triad traits of psychopathy and Machiavellianism were positively correlated with symptoms of ODD and CD, while the HEXACO traits agreeableness and honesty-humility were negatively correlated with such symptoms. Furthermore, it was found that both disruptive behavior disorders were associated with a unique set of temperament and personality correlates. More specifically, ODD symptoms were predicted by high psychopathy and low agreeableness and extraversion, whereas CD symptoms were predicted by high psychopathy and low honesty-humility, extraversion and emotionality.

The present study further examined the (unique) relations between temperament and personality traits and symptoms of ODD and CD in non-clinical adolescents. The study deviated in two ways from our previous investigation (11). To begin with, apart from the Dark Triad and HEXACO traits, we also measured two other relevant temperament and personality factors. The first one is impulsivity, which can be defined as the tendency to act quickly on the spur of the moment, without much thinking and consideration of the consequences (15). It is not only a key symptom of Attention-Deficit Hyperactivity Disorder (16)—a common comorbid condition of ODD and CD (3), but has also been put forward as the core of disruptive behavior disorders in general (17). The second trait is effortful control, which refers to individual differences in the ability to deploy attentional resources and to inhibit and activate behavioral responses with the purpose of self-regulation (18). Previous research has indicated that young people with low levels of effortful control are at increased risk for ODD and CD problems (19). By including these two traits, the current investigation aimed to provide a more comprehensive picture of temperament and personality traits involved in disruptive behavior problems of adolescents.

The temperament and personality traits included in this study originate from different theoretical models, but it can be assumed that there are meaningful relations between some traits of various accounts (8). For example, impulsivity can be expected to be negatively correlated with effortful control (20), and the Dark Triad traits are likely to show negative links to HEXACO agreeableness (21). In this study, we employ a tripartition of traits that was not empirically-derived but based on their qualitative nature: some were considered as “good” traits, which essentially help the person to adjust to the world and promote social behavior (i.e., honesty-humility, agreeableness, conscientiousness, openness to experience, and effortful control), some as “bad traits”, which have the potential to disturb the person's adaptation to the environment (i.e., emotionality and impulsivity), and others as “ugly” traits, which have a clear malevolent nature and directly fuel antisocial behavior (i.e., psychopathy, Machiavellianism, and narcissism) (22).

A further addition of the present study was that we examined a larger sample of adolescents, which made it possible to explore gender differences in the relations between temperament and personality traits and symptoms of disruptive behavior disorders. Previous research has indicated that ODD and CD are (somewhat) more prevalent in males as compared to females (23, 24). Moreover, significant gender differences have also been noted for various temperament and personality traits. For example, meta-analyses have indicated that males generally score higher on assertiveness, while females usually score higher on emotionality, sociability, agreeableness, and effortful control (25, 26). Given that disruptive behavior problems can also manifest themselves in different ways between both genders (e.g., males are more physically aggressive, have more school discipline problems, and more often engage in vandalism, whereas females are more likely to exhibit lying, truancy, running away, and prostitution) (3), it may well be the case that various temperament and personality traits make different contributions to symptoms of ODD and CD.

Following our previous study (11) and based on the extant literature (7, 8), we hypothesized ODD and CD to correlate negatively with the good traits of honesty-humility, agreeableness, conscientiousness, and effortful control, whereas positive correlations were anticipated with the bad trait of impulsivity and the ugly traits represented in the Dark Triad (in particular psychopathy and Machiavellianism). Our predictions regarding some other traits were less clear. For instance, extraversion is mostly considered to be a good trait because of its prosocial features and link with positive mood. However, it has also been pointed out that the high activity level associated with extraversion can cause social collisions and as such may prompt aggressive acts that are typical for disruptive behavior problems (10). A similar remark can be made regarding emotionality: on the hand, it seems plausible to assume that the aspect of irritability—which is incorporated in this trait—is positively related to (reactive) disruptive behavior (i.e., ODD), but there is also the notion that deficits in experiencing negative emotions (such as anxiety and guilt) may promote the occurrence of proactive, antisocial behavior (CD) (27)—which would be reflected by a negative correlation.

Furthermore, we expected that ODD and CD would each be associated with a unique set of temperament and personality predictors. Given that the present investigation assessed a broader range of temperament and personality traits (of which some were also expected to be significantly correlated), we had no well-defined hypothesis on what specific traits would make significant and unique contributions to both types of disruptive behavior problems. But on the basis of our previous study (11), we predicted that (low) agreeableness would play a dominant role in ODD, whereas the Dark Triad traits of psychopathy and Machiavellianism would be most powerful in predicting symptoms of CD. Finally, the examination of gender differences in the relation between temperament and personality traits and ODD and CD symptoms was explorative in nature.

## Method

### Participants and procedure

The participants in this study were 263 adolescents (109 males and 154 females) aged 12–18 years (mean age = 14.87 years, *SD* = 1.93). They were recruited between May 2022 and July 2022 via social media channels and schools. *A QR code was shared by common social media platforms (e.g., Facebook, WhatsApp), posters at public places frequently visited by young people (e.g., sporting clubs), and teachers at secondary schools. The code guided the young people to the online survey platform* Qualtrics. Participants were first presented with an information letter describing the goal, procedure, possible risks/discomforts, and benefits of the study, after which they were invited to fill in the consent form. Those aged 16 years and above gave their own consent to participate in the study, but for younger adolescents parental consent was also required. After obtaining informed consent, the participants were guided to the set of questionnaires, which were all in Dutch. Completion of the full survey took approximately 30 min. Of the initial sample of 369 participants, 106 did not finish the survey and their data were removed from the data set. This study was ethically evaluated and approved by the Ethics Review Committee of Psychology and Neuroscience (ERCPN) at Maastricht University, with reference number ERCPN-235_54_03_2021.

### Assessment

#### Temperament/personality traits

The HEXACO-Middle School Inventory (HEXACO-MSI) (28) is derived from the HEXACO Personality Inventory-Revised (29) and can be used in children and adolescents to assess the six basic traits of personality. More precisely, the 48 items (i.e., 8 items for each trait) measure: (1) honesty-humility (H), which includes characteristics such as sincerity, honesty, modesty, and faithfulness (e.g., “People should do what I say”, reversed item), (2) emotionality (E), which refers to interpersonal sensitivity and vulnerability and a tendency to respond in an overemotional way (e.g., “When a bad thing happens, I need someone who consoles me”), (3) extraversion (E), which covers features such as activeness, sociability, outgoingness, and talkativeness (e.g., “In general I feel happy about who I am”), (4) agreeableness (A), which has to do with prosocial features such as kindness, mildness, cooperativeness, and sympathy (e.g., “I am polite and quiet”), (5) conscientiousness (C), which reflects the tendency of being organized, disciplined, thorough, and precise (e.g., “I always doublecheck my homework to be sure that it is well done”), and (6) openness to experience (O), which pertains to characteristics of intellectuality, creativity, unconventionality, and innovativeness (e.g., “I would like to create a work of art, such as a novel, a song, or a painting”). Items are scored on a five-point scale with 1 = not at all true and 5 = very much true as anchors, and then combined to yield a score for each individual personality trait. A psychometric evaluation of the HEXACO-MSI has demonstrated that the scale is reliable in terms of both internal consistency and test-retest stability, and generally correlates in a theoretically meaningful way with another measure of personality traits (11, 28).

An age-downward version of the *Barratt Impulsiveness Scale-Short Form for Adolescents* (BIS-SFA) (30, 31) was used to measure individual differences in impulsivity. The scale consists of 15 items that can be allocated to three related domains of impulsivity, namely attention impulsivity (e.g., “I am restless during classes”), motor impulsivity (e.g., “I do things without thinking”), and non-planning (e.g., “I plan for the future”, reversed item). Items have to be rated on a four-point Likert scale with 1 = (almost) never, 2 = occasionally, 3 = often, and 4 = (almost) always. After recoding reversed items, scores can be combined into a total score, for which higher scores reflect higher levels of impulsivity. Previous research has indicated that the BIS-SF has good internal consistency, test-retest stability, and construct and concurrent validity (31, 32), and that similar positive psychometric properties have been obtained in adolescent populations from various countries including The Netherlands (33, 34).

The *Effortful Control Scale* (ECS) (35) is a self-report questionnaire that combines items of the Attention Control Scale (36) and the Early Adolescent Temperament Questionnaire (37) to assess various aspects of the regulative temperament characteristic of effortful control. In specific, the scale contains 15 items that cover attention focusing (e.g., “It is easy for me to really concentrate on homework problems”), attention shifting (e.g., “I can easily do two things at the same time”), and inhibitory control (e.g., “When someone tells me to stop doing something, it is easy for me to stop”). Respondents are asked to score the applicability of each item, using a four-point scale with 1 = not true, 2 = somewhat true, 3 = true, and 4 = very true. A total ECS score can be obtained by summing scores on all items. Previous studies have shown that the (Dutch) ECS is reliable (with a Cronbach's alpha of 0.73) (35) and there is also evidence for the validity of the scale (as demonstrated by a positive correlation of.67 with a measure of attention control) (38).

The *Dirty Dozen for Youth* (DD-Y) (39) is an age-downward version of Jonason and Webster's (40) concise measure of the Dark triad traits of psychopathy (e.g., “When I do something wrong, I feel little remorse”), Machiavellianism (e.g., “I try to influence others to get my way”), and narcissism (e.g., “I want others to admire me”). Each item has to be scored on a five-point Likert scale with anchors 1 = never true and 5 = (almost) always true. Scores are combined to yield a score for each individual “dark” trait. There is good evidence for the reliability and validity of the original Dirty Dozen (40, 41) and this is also true for the age-downward (Dutch) version of the instrument (39, 42).

As noted in the introduction, the temperament and personality traits as measured by all these instruments were allocated to three categories: good traits, which included HEXACO-MSI honesty-humility, extraversion, agreeableness, conscientiousness, openness to experience, and ECS effortful control; bad traits, which included HEXACO-MSI emotionality and BIS-SFA impulsivity; and ugly traits, which included DD-Y psychopathy, Machiavellianism, and narcissism (22).

### Disruptive behavior problems

Adolescents' disruptive behavior problems were measured with DSM-based problem scales of ODD and CD of the *Youth Self-Report* (YSR) (43). The ODD scale consists of five items (e.g., “I argue a lot” and “I am disobedient at home”), while the CD scale contains 14 items (e.g., “I am cruel or mean to people” and “I am truant or skip school”). Young people are asked to rate the applicability of various items, using a three-point scale with 0 = not at all, 1 = a little or sometimes, and 2 = clearly or often. For both scales, a total score can be obtained by summing ratings on relevant items. There is abundant evidence for the psychometric properties of the YSR (44) and a substantial part of these data have also been collected in The Netherlands (45). Furthermore, a study by Burt et al. (46) has indicated that the ODD and CD scales of this instrument represent related but distinct types of disruptive behavior problems.

### Statistical analyses

The Statistical Package of Social Sciences was used to conduct the data analyses. First, we calculated descriptive statistics (means and standard deviations) and reliability coefficients (Cronbach's alphas) for all questionnaires and evaluated gender differences for these measures by means of independent samples *t*-tests. Then, we computed correlation coefficients among all questionnaires. Finally, we performed regression analyses with personality traits as predictor variables and either YSR ODD problems or YSR CD problems as the dependent variable. As we were interested in finding the “best” predictors of both types of disruptive behavior problems, the stepwise method was used in the regression analyses. We also adopted a hierarchical approach in that the first analysis only included “good traits” as predictors, whereas “bad traits” and “ugly traits” were added in respectively the second and third model. In the regression analyses, multicollinearity diagnostics were checked: the results showed the variance inflation factor (VIF) and tolerance values were all within an acceptable range (VIF: .72–1.00; tolerance 1.00–1.37). To explore gender effects, correlation and regression analyses were also conducted for males and females separately.

## Results

### General findings

Before discussing the main results of this study, some general findings should be addressed. First of all, independent samples *t*-tests revealed statistically significant gender differences for a number of measures. As can be seen in Table 1, females scored higher on emotionality [*t*(261) = 6.13, *p* < 0.001] and openness to experience [*t*(261) = 2.63, *p* < 0.01], whereas males displayed higher levels of extraversion [*t*(261) = 4.11, *p* < 0.001], agreeableness [*t*(261) = 2.70, *p* < 0.01], psychopathy [*t*(261) = 4.26, *p* < 0.001], and Machiavellianism [*t*(261) = 2.83, *p* < 0.01]. Second, questionnaires generally displayed satisfactory internal consistency, with most Cronbach's alpha values being well above.70 (see Table 1). There were two exceptions to this rule: HEXACO-MSI emotionality and YSR ODD showed alphas of.62 and.66, respectively, but given the fact that these scales consisted of a restricted number of items, these values can still be interpreted as acceptable. Third and finally, comparison of the mean scores on the YSR scales with normative data of this measure revealed that respectively 8.4% and 0.4% of the adolescents scored above the subclinical and clinical cut-off of ODD, whereas 12.2% and 2.7% scored above the subclinical and clinical cut-off for CD. These YSR data confirmed the non-clinical nature of the present sample.

**Table 1:** Descriptive statistics for various questionnaires: mean scores (standard deviations), gender differences, and reliability coefficients.

	Total sample (*N* = 263)	Males (*n *= 109)	Females (*n* = 154)	Cronbach's *α*
HEXACO-MSI				
Honesty-humility	32.96 (5.26)	32.43 (5.41)	33.34 (5.14)	.74
Emotionality	22.31 (5.00)	20.20 (4.82)	23.80 (4.59)**	.62
eXtraversion	27.43 (6.68)	29.39 (6.65)	26.05 (6.37)**	.83
Agreeableness	25.35 (5.51)	26.43 (5.02)	24.59 (5.73)^*^	.70
Conscientiousness	24.13 (6.16)	23.49 (5.72)	24.58 (6.44)	.76
Openness	20.26 (7.02)	18.96 (6.18)	21.18 (7.44)^*^	.76
BIS-SF				
Impulsivity	33.35 (6.58)	33.72 (6.39)	33.10 (6.73)	.83
ECS				
Effortful control	41.37 (6.17)	41.42 (6.55)	41.34 (5.90)	.72
DD-Y				
Psychopathy	7.42 (2.89)	8.29 (2.81)	6.80 (2.80)**	.70
Machiavellianism	7.14 (2.72)	7.70 (2.67)	6.75 (2.69)^*^	.76
Narcissism	8.14 (3.02)	8.17 (2.97)	8.12 (3.07)	.75
YSR				
ODD problems	2.23 (1.97)	2.09 (1.90)	2.32 (2.02)	.66
CD problems	3.81 (3.53)	4.17 (3.09)	3.56 (3.80)	.80

HEXACO-MSI, HEXACO-Middle School Inventory; BIS-SFA, Barratt Impulsiveness Scale, Short Form for Adolescents; ECS, Effortful Control Scale; DD-Y, Dirty Dozen for Youth; YSR, Youth Self-Report; ODD, oppositional defiant disorder; CD, conduct disorder.

* Significant gender difference at *p* < 0.001.

### Correlations among temperament/personality traits and disruptive behavior problems

Table 2 shows correlations among all questionnaires. The correlations for the total sample (which were controlled for gender) are presented below the diagonal. First and foremost, it was found that temperament and personality traits were associated in the predicted way with ODD and CD symptoms. More precisely, the good traits of honesty-humility, agreeableness, conscientiousness, and effortful control were all negatively (*r*'s between −0.27 and −0.61), while the bad trait of impulsivity and the ugly traits of psychopathy, Machiavellianism, and narcissism were all positively (*r*'s between 0.25 and 0.58) correlated with these disruptive behavior problems, with most correlations being of a medium to large effect size. Note also that the correlations between emotionality and extraversion and symptoms of ODD and CD were all negative (*r*'s between −0.12 and −0.20). Although the magnitude of these correlations was fairly small, this finding indicates that higher levels of emotionality and extraversion were (to some extent) associated with lower levels of disruptive behavior problems.

**Table 2:** Correlations among scores on various personality traits and disruptive behavior problems.

	(1)	(2)	(3)	(4)	(5)	(6)	(7)	(8)	(9)	(10)	(11)	(12)	(13)
HEXACO-MSI													
(1) Honesty-humility		.25/.08	.15/.02	**.52**/**.43**	.21/.15	.28/.17	**−.38**/**−.33**	.24/.27	**−.41**/**−.43**	**−.51**/**−.64**	−.**44**/**−.46**	−.27/**−.38**	**−.46**/**−.49**
(2) Emotionality	.18^*^		−.28/−.07	.14/**.31**	.06/.18	.17/.07	−.06/−.25	−.04/−.11	**−.39**/**−.33**	−.21/−.05	.05/.20	−.08/−.23	−.20/−.14
(3) eXtraversion	.05	−.23*^**^		.12/.13	.17/−.07	−.05/−.27	−.05/−.10	.18/.34	.15/−.04	−.02/−.12	−.11/−.01	−.22/−.16	−.16/−.16
(4) Agreeableness	.44^**^	.16^*^	.16^*^		.28/**.33**	.26/.03	**−.45**/**−.54**	**.50**/**.39**	**−.34**/**−.42**	**−.44**/**−.45**	**−.35**/−.26	**−.59**/**−.62**	**−.50**/**−.4**1
(5) Conscientiousness	.18^*^	.15^*^	.00	.29^**^		.21/.17	**−.60**/**−.65**	**.41**/**.28**	−.03/−.25	−.04/−.17	.02/.03	−.32/−.27	−.29/−.25
(6) Openness	.22^**^	.15^*^	−.22^**^	.08	.20^*^		−.28/−.25	.08/−.05	−.11/−.17	−.08/−.02	.20/.22	−.19/−.02	−.08/−.13
BIS-SFA													
(7) Impulsivity	−.35^**^	−.18^*^	−.06	−.49^**^	−.63^**^	−.26^**^		**−.60**/**−.47**	.19/**.44**	.26/**.37**	.16/.08	**.46**/**.49**	**.40**/**.44**
ECS													
(8) Effortful control	.25^**^	−.08	.26^**^	.43^**^	.33^**^	.00	−.53^**^		.02/−.21	−.25/**−.40**	−.29/**−.33**	**−.53**/**−.39**	−.31/**−.39**
DD-Y													
(9) Psychopathy	−.42^**^	−.41^**^	.10	−.33^**^	−.18^*^	−.18^*^	.34^**^	−.10		.**49**/**.57**	.20/.17	.29/**.59**	**.39**/**.67**
(10) Machiavellianism	−.59^**^	−.17^*^	−.03	−.40^**^	−.14^*^	−.07	.33^**^	−.32^**^	.55^**^		**.47**/**.55**	.30/**.59**	**.49**/**.62**
(11) Narcissism	−.45^**^	.12^*^	−.05	−.29^**^	.02	.21^*^	.11	−.31^**^	.18^*^	.51^**^		.13/**.32**	.32/**.29**
YSR													
(12) ODD problems	−.33^**^	−.13^*^	−.20^*^	−.61^**^	−.28^**^	−.07	.48^**^	−.45^**^	.44^**^	.45^**^	.25^**^		**.58**/**.74**
(13) CD problems	−.48^**^	−.18^*^	−.13^*^	−.42^**^	−.27^**^	−.13^*^	.42^**^	−.36^**^	.57^**^	.58^**^	.30^**^	.67^**^	

Below the diagonal: correlations for the total sample, and above the diagonal: correlations for males (left) and females (right) separately. Total sample: *N* = 263, males: *n* = 109, females: *n* = 154. HEXACO-MSI, HEXACO-Middle School Inventory; BIS-SFA, Barratt Impulsiveness Scale, Short Form for Adolescents; ECS, Effortful Control Scale; DD-Y, Dirty Dozen for Youth; YSR, Youth Self-Report; ODD, oppositional defiant disorder; CD, conduct disorder. Gender-specific correlations printed in bold were significant at *p* < .001.

* For partial correlations based on the total *N*: *p* < 0.05.

** For partial correlations based on the total *N*: *p* < .001.

Correlations among temperament and personality traits also showed the to-be-expected pattern. That is, the good traits of honesty-humility and agreeableness were positively correlated (*r *= 0.44), and each of them were at their turn negatively correlated with the three Dark Triad members (all *r*'s between −0.29 and −0.59). The Dark Triad traits were also positively correlated, although the correlation between psychopathy and narcissism was rather small (*r* = 0.18). Furthermore, the good traits of conscientiousness and effortful control were positively intercorrelated (*r* = 0.33) and both were substantially, negatively correlated with the bad trait of impulsivity (*r*'s being −0.63 and −0.53, respectively). Finally, a positive correlation was noted between ODD and CD symptoms (*r* = 0.67).

Correlations were also computed for both genders separately. The results are displayed above the diagonal in Table 2. The main conclusion of these analyses was that although the size of the correlations somewhat varied between males and females, the general pattern of the correlations found between temperament and personality traits and ODD and CD symptoms was quite similar to that documented for the total sample.

### Unique contributions of good, bad, and ugly traits to ODD/CD

#### ODD

The results of the hierarchical, stepwise regression analyses with ODD symptoms as the dependent variable and various sets of temperament and personality traits (i.e., model 1: good traits, model 2: good and bad traits, and model 3: good, bad and ugly traits) as the predictors are displayed in Table 3. As can be seen, when predicting ODD symptoms from good traits (model 1), agreeableness (step 1) and effortful control (step 2) entered as predictors, together accounting for 41% of the total variance. Both beta values were negative, which indicated that higher levels of these good traits were associated with lower levels of ODD. When considering good and bad traits as predictors of ODD (model 2), agreeableness (step 1) and effortful control (step 2) remained significant predictors, but now impulsivity entered in the model on the third step, accounting for an extra 2% of the variance. Note that the beta value of impulsivity was positive, implying that higher levels of this trait were associated with higher levels of ODD symptoms. In the third model in which good, bad, and ugly traits were possible predictors of ODD, agreeableness (step 1) and effortful control (step 3) were again found to make significant contributions alongside psychopathy (step 2) and extraversion (step 4). The beta value for psychopathy was positive, which means that higher levels of this Dark Triad trait were associated with higher symptom levels of ODD, whereas the beta value for extraversion was negative, which implies that higher levels of this good trait were accompanied by lower levels of this type of disruptive behavior. In total, the third model explained 49% of the variance in ODD symptoms.

**Table 3:** Results of the stepwise regression analyses with self-reported personality features as the predictors and YSR ODD problems in the total sample as the dependent variable.

	Beta	SE	ß	(Δ)*R*^2^
Model 1				
1. HEXACO-MSI Agreeableness^MF^	−.22	.02	−.61^**^	0.37^**^
2. ECS Effortful control^MF^	−.07	.02	−.23^**^	0.04^**^
Final model: *F*(2,260) = 91.61, *p *< .001				0.41^**^
Model 2				
1. HEXACO-MSI Agreeableness^MF^	−.22	.02	−.61^**^	0.37^**^
2. ECS Effortful control^M^	−.07	.02	−.23^**^	0.04^**^
3. BIS-SFA Impulsivity^F^	.05	.02	.17^*^	0.02^*^
Final model: *F*(3,259) = 65.37, *p *< .001				0.43^**^
Model 3				
1. HEXACO-MSI Agreeableness^MF^	−.22	.02	−.61^**^	0.37^**^
2. DD-Y Psychopathy^MF^	.18	.03	.27^**^	0.06^**^
3. ECS Effortful control^M^	−.08	.02	−.24^**^	0.05^**^
4. HEXACO-MSI Extraversion^M^	−.03	.01	−.11^*^	0.01^*^
Final model: *F*(4,258) = 62.06, *p *< .001				0.49^**^

Model 1 only included good traits, model 2 good and bad traits, and model 3 good, bad, and ugly traits. *N* = 263. YSR, Youth Self-Report; ODD,  oppositional defiant disorder; HEXACO-MSI,  HEXACO-Middle School Inventory; ECS,  Effortful Control Scale; BIS-SFA,  Barratt Impulsiveness Scale, Short Form for Adolescents; DD-Y,  Dirty Dozen for Youth. Good traits: HEXACO-MSI honesty-humility, extraversion, agreeableness, conscientiousness, openness to experience, and ECS effortful control; bad traits: HEXACO-MSI emotionality and BIS-SFA impulsivity; ugly traits: DD-Y psychopathy, Machiavellianism, and narcissism. Superscripts M and F indicate whether the pertinent variable made a significant contribution in the regression models that were conducted for males and females separately (see Supplementary Tables S1, S2).

* *p* < 0.05.

** *p* < 0.001.

The regression analyses predicting ODD symptoms in both genders separately (Supplementary Tables S1, S2) revealed that the same traits were included in models 1 and 2 as in the analyses of the total sample. This changed when the ugly traits were added as possible predictors (i.e., model 3). In males, ODD symptoms were predicted by agreeableness (step 1), effortful control (step 2), psychopathy (step 3), extraversion (step 4), and narcissism (step 5). In females, agreeableness (step 1), psychopathy (step 2), and Machiavellianism (step 3) were found to be significant, unique predictors of this type of disruptive behavior. In both genders, a considerable proportion of the variance in ODD symptoms was explained, namely 49% in males and 56% in females. In almost all cases, the direction of the beta values was as expected, with good traits being negatively and bad and ugly traits being positively related to ODD symptoms. The one exception was narcissism, which made a negative contribution to ODD in males, which suggests that there are some unique features in this Dark Triad member that are associated with lower levels of this type of disruptive behavior.

#### CD

The results of the regression analysis with CD symptoms as the dependent variable are shown in Table 4. In model 1 in which CD symptoms were predicted from good traits, honesty-humility (step 1), effortful control (step 2), and agreeableness (step 3) made significant, unique contributions, together explaining 31% of the total variance. When adding the bad traits (model 2), impulsivity was also included, which increased the explained variance with an extra 2%. With the addition of the ugly traits (model 3), the percentage of explained variance further increased with 17%. Effortful control and honesty-humility were retained in this model, but the strongest predictors of CD symptoms appeared to be Machiavellianism and psychopathy. Furthermore, extraversion and conscientiousness were also included in this model, accounting for a small but unique and statistically significant proportion of the variance: their negative beta values pointed out that higher levels of these good traits were associated with lower levels of CD symptoms.

**Table 4:** Results of the stepwise regression analyses with self-reported personality features as the predictors and YSR CD problems in the total sample as the dependent variable.

	Beta	SE	*β*	(Δ)*R*^2^
Model 1				
1. HEXACO-MSI Honesty-humility^MF^	−.32	.04	−.48^**^	0.23^**^
2. ECS Effortful control^F^	−.14	.03	−.25^**^	0.06^**^
3. HEXACO-MSI Agreeableness^MF^	−.12	.04	−.18^*^	0.02^*^
Final model: *F*(3,259) = 39.38, *p *< .001				0.31^**^
Model 2				
1. HEXACO-MSI Honesty-humility^MF^	−.32	.04	−.48^**^	0.23^**^
2. BIS-SFA Impulsivity^F^	.16	.03	.29^**^	0.07^**^
3. HEXACO-MSI Agreeableness^M^	−.10	.04	−.16^*^	0.02^*^
4. ECS Effortful control^F^	−.07	.04	−.12^*^	0.01^*^
Final model: *F*(4,258) = 32.15, *p *< .001				0.33^**^
Model 3				
1. DD-Y Machiavellianism^MF^	.75	.07	.58^**^	0.33^**^
2. DD-Y Psychopathy^F^	.44	.07	.36^**^	0.09^**^
3. ECS Effortful control^F^	−.13	.03	−.22^**^	0.04^**^
4. HEXACO-MSI Honesty-humility	−.10	.04	−.15^*^	0.01^*^
5. HEXACO-MSI Extraversion	−.06	.03	−.11^*^	0.01^*^
6. HEXACO-MSI Conscientiousness^M^	−.06	.03	−.10^*^	.01^*^
Final model: *F*(6,256) = 42.06, *p *< .001				0.50^**^

*N* = 263. YSR,  Youth Self-Report; CD,  conduct disorder; HEXACO-MSI,  HEXACO-Middle School Inventory; ECS,  Effortful Control Scale; BIS-SFA,  Barratt Impulsiveness Scale, Short Form for Adolescents; DD-Y,  Dirty Dozen for Youth. Good traits: HEXACO-MSI honesty-humility, extraversion, agreeableness, conscientiousness, openness to experience, and ECS effortful control; bad traits: HEXACO-MSI emotionality and BIS-SFA impulsivity; ugly traits: DD-Y psychopathy, Machiavellianism, and narcissism. Superscripts M and F indicate whether the pertinent variable made a significant contribution in the regression models that were conducted for males and females separately (see Supplementary Tables S3, S4).

* *p* < 0.05.

** *p* < 0.001.

The gender-specific analyses (Supplementary Tables S3, S4) revealed that model 1, which focused on the good traits as predictors of CD, was largely comparable for males and females, with honesty-humility and agreeableness being the main predictors In females, effortful control was also included as a significant predictor in model 1. When adding the bad traits, impulsivity entered in the model, but this was only the case in females. Results for males and females became quite different when adding the ugly traits as predictors (model 3). In males, CD symptoms were predicted by agreeableness (step 1), Machiavellianism (step 2), and conscientiousness (step 3), together accounting for 37% of the total variance. In females, this type of disruptive behavior was predicted by psychopathy (step 1), Machiavellianism (step 2), and effortful control (step 3), and jointly these predictors explained 57% of the variance in CD symptoms. In all cases, the direction of the beta values was as anticipated, with good traits being negatively and bad and ugly traits being positively related to symptoms of CD.

## Discussion

The aim of the present study was to provide a comprehensive picture of self-reported temperament and personality correlates of disruptive behavior problems in a non-clinical sample of male and female adolescents. The results first of all showed that the correlations between temperament and personality traits and symptoms of ODD and CD were largely as anticipated. That is, young people who display higher levels of disruptive behavior problems are characterized by lower levels of sincerity, fairness, modesty, and faithfulness (i.e., honesty-humility) (47, 48), lower levels of kindness, mildness, cooperativeness, and sympathy (agreeablesness) (49, 50), lower levels of organization, discipline, thoroughness, and precision (conscientiousness) (51, 52), and lower levels of regulative abilities of attention control, attention shifting, and inhibitory control (effortful control) (19, 53). Further, in keeping with previous studies, it was found that adolescents with higher levels of disruptive behavior problems can be typified by a stronger tendency to act impulsively (17, 54) and higher levels of malevolent personality features covered by the Dark Triad (39, 55).

Second, symptoms of ODD and CD were positively intercorrelated, which confirms that the prototypical antagonism and aggressive/delinquent acts that are reflected by these problems represent related types of disruptive behavior (56). However, there are also some marked distinctions between ODD and CD. For example, the behaviors associated with ODD are of a less severe nature in terms of violations of morality and other people's integrity than those observed in CD (3). Furthermore, it has been demonstrated that problems of emotional dysregulation are more prominent in ODD than in CD (57). In line with these notions, the results showed that agreeableness and effortful control were important predictor variables of ODD, whereas the model predicting symptoms of CD symptoms was mainly dominated by lack of honesty-humlity and the Dark Triad members of Machiavellianism and psychopathy.

Third, exploration of gender differences in the temperament and personality correlates of disruptive behavior problems revealed that—in both males and females—symptoms of ODD were mainly predicted by agreeableness and psychopathy. Contributions of other variables varied between both genders but their percentages of explained variance were relatively small. More clear differences were noted in the regression analysis predicting symptoms of CD, and this was in particular the case when the ugly traits were added to the model. In males, agreeableness emerged as the most important predictor of CD symptoms, followed by Machiavellianism, and conscientiousness, whereas in females this type of disruptive behavior problems was predominantly predicted by psychopathy, after which Machiavellianism and effortful control entered into the model. Further, it was also notable that in females various temperament and personality traits explained much more variance in CD symptoms than in males (i.e., 57% vs. 37%). These findings can be explained by the fact that males and females differ in terms of temperament and personality traits (25, 26, 58), phenomenological expressions of disruptive behavior (59, 60), as well as their willingness to report openly on immoral and antisocial tendencies and actions (61, 62). However it may be, the results are well in line with a previous study by Dinic and Wertag (63) who explored gender differences in HEXACO and Dark Triad correlates of aggression, and also found that this type of disruptive behavior was associated with different personality profiles in males and females (50, 64).

The present study also yielded a number of additional findings that deserve some brief comment. First, emotionality, which in the literature has been considered as a “bad” trait—mainly because of its positive relationship with psychopathology of an internalizing nature (65), generally showed small, but statistically significant, negative correlations with both types of disruptive behavior problems (only in males, the correlation with ODD problems was not significant). This suggests that this trait is at least to some extent shielding young persons against ODD and CD problems, which is in line with the notion that unemotional characters are associated with the most persistent and deviant behaviors, simply because they lack anxiety and guilt and are insensitive to the negative consequences that their antagonistic and antisocial actions may have for others (66). Admittedly, in the regression models, emotionality never emerged as a unique predictor of disruptive behavior problems, but it seems most plausible that low emotionality was covered by the dark trait of psychopathy, of which deficient emotional responses is a defining feature (27). In line with this explanation, the present study indeed found a quite substantial negative correlation between emotionality and psychopathy. Second, although there are scholars who have found that extraversion is positively associated with disruptive behavior problems (10), the results of the present study indicated that this basic personality trait was negatively correlated with symptoms of ODD and CD, which confirmed its status of being a good trait within the context of psychopathology (65). Even in the regression models, extraversion was included as a negative predictor, accounting for a small but significant proportion of the variance. This indicates that this personality trait incorporates some unique positive features that are difficult to reconcile with disruptive behavior problems which are not covered by other temperament/personality traits. Third, as expected the Dark Triad member of narcissism correlated positively with symptoms of ODD and CD. However, in males, once controlling for other personality and temperament traits, narcissism made a small but significant *negative* contribution to symptoms of ODD. Previous studies have indicated that—besides dark features—narcissism also harbors positive characteristics (e.g., mental toughness, high self-esteem) (67) that may explain the negative association with symptoms of ODD once the evil elements are filtered out.

It should be acknowledged that the present investigation suffered from a number of limitations. To begin with, the study was conducted in a non-clinical sample of adolescents that was recruited via schools and through advertisements in social media. As such we could not establish the exact response rate of this study and so it remains unclear to what the results can be generalized to the general population of young people and/or to clinically referred young people who are actually diagnosed with the disruptive behavior disorders of ODD and CD. To increase the sample size of the study, a mixed survey completion method (i.e., online vs. offline) was used and it is possible that the different settings in which the questionnaires were fillied in may also have been a threat to the reliability and validity of the measurements (e.g., because of a distracting environment, motivational issues). Furthermore, the study merely relied on young people's self-report. In particular in the case of problems of an externalizing nature (such as ODD and CD), other informants such as parents and teachers are preferred because young people themselves typically underreport on these types of problems (68). A comparable mechanism may be operating in the self-report of Dark Triad traits. Typically, these traits are associated with dishonesty and deceit, and so persons who score higher on psychopathy, Machiavellianism, and narcissism are probably less inclined to report openly on the “ugly” sides of their personality and behavior (69). Another shortcoming pertains to the cross-sectional nature of the study. Although the regression analyses were conducted in such way that personality and temperament traits were the predictors of disruptive behavior symptoms, caution must be exercised regarding the interpretation of these correlational data in terms of cause-effect relations. A final demerit concerns the use of subscales of the Youth Self-Report as an index of ODD and CD symptomatology. While this measure is generally seen as an effective screening instrument, it is also true that it contains a rather limited set of items. For example, the ODD subscale is only covered by five items which showed moderate internal consistency. There are more specialized and more reliable and valid scales for assessing these disruptive behavior problems (70), and preferably these measures should be employed in future studies.

In conclusion, the present study made an attempt to provide a comprehensive picture of self-reported temperament and personality traits as correlates of disruptive behavior problems in male and female adolescents. In general, the correlational analysis yielded the to-be-expected pattern, indicating that good traits (in particular, honesty-humility, agreeableness, and regulative trait of effortful control) were negatively associated, while bad and ugly traits (especially impulsivity and the Dark Triad traits of psychopathy and Machiavellianism) were positively associated with symptoms of ODD and CD. Regression analyses indicated that ODD and CD symptoms were each associated with a unique set of temperament and personality characteristics, and also pointed out that these predictors were somewhat different for male and female adolescents. These findings indicate that various theoretical frameworks on temperament and personality are relevant for understanding the etiology of disruptive behavior problems, implying that further studies on this topic should take good as well as bad and ugly traits into account. Further, greater consideration should be given to gender differences in the specific traits underlying ODD and CD in the adolescent years, and the implications that this has for the assessment of such problems in research as well as in clinical practice.

## Data Availability

The raw data supporting the conclusions of this article will be made available by the authors, without undue reservation.
