# Supplementary material for: The good, the bad, and the ugly: a comprehensive study of temperament and personality traits as correlates of self-reported disruptive behavior problems in male and female adolescents
Source: Front Child Adolesc Psychiatry. 2023 May 22;2:1173272. doi: 10.3389/frcha.2023.1173272 (PMC11731598; doi:10.3389/frcha.2023.1173272)
Supplement: Supplementary file 1 [file Table1.docx]

**Supplementary materials**

**Gender-specific regression analyses**

**Table S1**

Results of the stepwise regression analyses with self-reported personality features as the predictors and YSR **ODD problems** in **males** as the dependent variable. Model 1 only included *good* traits, model 2 *good and bad* traits, and model 3 *good, bad, and ugly* traits.

|  | Beta | SE | ß | (∆)*R*^2^ |
| --- | --- | --- | --- | --- |
|  |  |  |  |  |
|  |  |  |  |  |
|  |  |  |  |  |
| Model 1 |  |  |  |  |
| 1. HEXACO-MSI Agreeableness | -.22 | .03 | -.59** | .35** |
| 2. ECS Effortful control | -.09 | .03 | -.31** | .07** |
|  |  |  |  |  |
|  |  |  |  |  |
| Final model: *F*(2,106) = 38.53, *p* < .001 |  |  |  | .42** |
|  |  |  |  |  |
| Model 2 |  |  |  |  |
| 1. HEXACO-MSI Agreeableness | -.22 | .03 | -.59** | .35** |
| 2. ECS Effortful control | -.09 | .03 | -.31** | .07** |
|  |  |  |  |  |
|  |  |  |  |  |
| Final model: *F*(2,106) = 38.53, *p* < .001 |  |  |  | .42** |
|  |  |  |  |  |
| Model 3 |  |  |  |  |
| 1. HEXACO-MSI Agreeableness | -.22 | .03 | -.59** | .35** |
| 2. ECS Effortful control | -.09 | .03 | -.31** | .07** |
| 3. DD-Y Psychopathy | .12 | .05 | .18* | .03* |
| 4. HEXACO-MSI Extraversion | -.04 | .02 | -.15* | .02* |
| 5. DD-Y Narcissism | -.11 | .05 | -.17* | .02* |
|  |  |  |  |  |
|  |  |  |  |  |
| Final model: *F*(5,103) = 20.13, *p* <.001 |  |  |  | .49** |
|  |  |  |  |  |

*Note*. *N* = 109. YSR = Youth Self-Report, ODD = Oppositional Defiant Disorder, HEXACO-MSI = HEXACO-Middle School Inventory, ECS = Effortful Control Scale, BIS-SFA = Barratt Impulsiveness Scale, Short Form for Adolescents, DD-Y = Dirty Dozen for Youth. Good traits: HEXACO-MSI honesty-humility, extraversion, agreeableness, conscientiousness, openness to experience, and ECS effortful control; bad traits: HEXACO-MSI emotionality and BIS-SFA impulsivity; ugly traits: DD-Y psychopathy, Machiavellianism, and narcissism. * *p* < .05, ** *p* < .001

**Table S2**

Results of the stepwise regression analyses with self-reported personality features as the predictors and YSR **ODD problems** in **females** as the dependent variable. Model 1 only included *good* traits, model 2 *good and bad* traits, and model 3 *good, bad, and ugly* traits.

|  | Beta | SE | ß | (∆)*R*^2^ |
| --- | --- | --- | --- | --- |
|  |  |  |  |  |
|  |  |  |  |  |
|  |  |  |  |  |
| Model 1 |  |  |  |  |
| 1. HEXACO-MSI Agreeableness | -.22 | .02 | -.62** | .39** |
| 2. ECS Effortful control | -.06 | .02 | -.17* | .02* |
|  |  |  |  |  |
|  |  |  |  |  |
| Final model: *F*(2,151) = 52.55, *p* < .001 |  |  |  | .41** |
|  |  |  |  |  |
| Model 2 |  |  |  |  |
| 1. HEXACO-MSI Agreeableness | -.22 | .02 | -.62** | .39** |
| 2. BIS-SFA Impulsivity | .07 | .02 | .22* | .04* |
|  |  |  |  |  |
|  |  |  |  |  |
| Final model: *F*(2,151) = 54.75, *p* < .001 |  |  |  | .43** |
|  |  |  |  |  |
| Model 3 |  |  |  |  |
| 1. HEXACO-MSI Agreeableness | -.22 | .02 | -.62** | .39** |
| 2. DD-Y Psychopathy | .29 | .05 | .40** | .13** |
| 3. DD-Y Machiavellianism | .19 | .05 | .25** | .04** |
|  |  |  |  |  |
|  |  |  |  |  |
| Final model: *F*(3,150) = 62.18, *p* <.001 |  |  |  | .56** |
|  |  |  |  |  |

*Note*. *N* = 154. YSR = Youth Self-Report, ODD = Oppositional Defiant Disorder, HEXACO-MSI = HEXACO-Middle School Inventory, ECS = Effortful Control Scale, BIS-SFA = Barratt Impulsiveness Scale, Short Form for Adolescents, DD-Y = Dirty Dozen for Youth. Good traits: HEXACO-MSI honesty-humility, extraversion, agreeableness, conscientiousness, openness to experience, and ECS effortful control; bad traits: HEXACO-MSI emotionality and BIS-SFA impulsivity; ugly traits: DD-Y psychopathy, Machiavellianism, and narcissism. * *p* < .05, ** *p* < .001

**Table S3**

Results of the stepwise regression analyses with self-reported personality features as the predictors and YSR **CD problems** in **males** as the dependent variable. Model 1 only included *good* traits, model 2 *good and bad* traits, and model 3 *good, bad, and ugly* traits.

|  | Beta | SE | ß | (∆)*R*^2^ |
| --- | --- | --- | --- | --- |
|  |  |  |  |  |
|  |  |  |  |  |
|  |  |  |  |  |
| Model 1 |  |  |  |  |
| 1. HEXACO-MSI Agreeableness | -.31 | .05 | -.50** | .25** |
| 2. HEXACO-MSI Honesty-humility | -.16 | .05 | -.28* | .06* |
|  |  |  |  |  |
|  |  |  |  |  |
| Final model: *F*(2,106) = 23.37, *p* < .001 |  |  |  | .31** |
|  |  |  |  |  |
| Model 2 |  |  |  |  |
|  |  |  |  |  |
| 1. HEXACO-MSI Agreeableness | -.31 | .05 | -.50** | .25** |
| 2. HEXACO-MSI Honesty-humility | -.16 | .05 | -.28* | .06* |
|  |  |  |  |  |
|  |  |  |  |  |
| Final model: *F*(2,106) = 23.37, *p* < .001 |  |  |  | .31** |
|  |  |  |  |  |
|  |  |  |  |  |
| Model 3 |  |  |  |  |
| 1. HEXACO-MSI Agreeableness | -.31 | .05 | -.50** | .25** |
| 2. DD-Y Machiavellianism | .39 | .10 | .33** | .09** |
| 3. HEXACO-MSI Conscientiousness | -.11 | .04 | -.20* | .03* |
|  |  |  |  |  |
|  |  |  |  |  |
| Final model: *F*(3,105) = 20.85, *p* <.001 |  |  |  | .37** |
|  |  |  |  |  |

*Note*. *N* = 109. YSR = Youth Self-Report, CD = Conduct Disorder, HEXACO-MSI = HEXACO-Middle School Inventory, ECS = Effortful Control Scale, BIS-SFA = Barratt Impulsiveness Scale, Short Form for Adolescents, DD-Y = Dirty Dozen for Youth. Good traits: HEXACO-MSI honesty-humility, extraversion, agreeableness, conscientiousness, openness to experience, and ECS effortful control; bad traits: HEXACO-MSI emotionality and BIS-SFA impulsivity; ugly traits: DD-Y psychopathy, Machiavellianism, and narcissism. * *p* < .05, ** *p* < .001

**Table S4**

Results of the stepwise regression analyses with self-reported personality features as the predictors and YSR **CD problems** in **females** as the dependent variable. Model 1 only included *good* traits, model 2 *good and bad* traits, and model 3 *good, bad, and ugly* traits.

|  | Beta | SE | ß | (∆)*R*^2^ |
| --- | --- | --- | --- | --- |
|  |  |  |  |  |
|  |  |  |  |  |
|  |  |  |  |  |
| Model 1 |  |  |  |  |
| 1. HEXACO-MSI Honesty-humility | -.36 | .05 | -.49** | .24** |
| 2. ECS Effortful control | -.18 | .05 | -.28** | .07** |
| 3. HEXACO-MSI Agreeableness | -.11 | .05 | -.17* | .02* |
|  |  |  |  |  |
|  |  |  |  |  |
| Final model: *F*(3,150) = 25.29, *p* < .001 |  |  |  | .32** |
|  |  |  |  |  |
| Model 2 |  |  |  |  |
| 1. HEXACO-MSI Honesty-humility | -.36 | .05 | -.49** | .24** |
| 2. BIS-SFA Impulsivity | .17 | .04 | .31** | .08** |
| 3. ECS Effortful control | -.12 | .05 | -.19* | .03* |
|  |  |  |  |  |
|  |  |  |  |  |
| Final model: *F*(3,150) = 27.14, *p* < .001 |  |  |  | .35** |
|  |  |  |  |  |
| Model 3 |  |  |  |  |
| 1. DD-Y Psychopathy | .91 | .08 | .67** | .45** |
| 2. DD-Y Machiavellianism | .50 | .10 | .36** | .09** |
| 3. ECS Effortful control | -.12 | .04 | -.18* | .03* |
|  |  |  |  |  |
|  |  |  |  |  |
| Final model: *F*(3,150) = 65.02, *p* <.001 |  |  |  | .57** |
|  |  |  |  |  |

*Note*. *N* = 154. YSR = Youth Self-Report, CD = Conduct Disorder, HEXACO-MSI = HEXACO-Middle School Inventory, ECS = Effortful Control Scale, BIS-SFA = Barratt Impulsiveness Scale, Short Form for Adolescents, DD-Y = Dirty Dozen for Youth. Good traits: HEXACO-MSI honesty-humility, extraversion, agreeableness, conscientiousness, openness to experience, and ECS effortful control; bad traits: HEXACO-MSI emotionality and BIS-SFA impulsivity; ugly traits: DD-Y psychopathy, Machiavellianism, and narcissism. * *p* < .05, ** *p* < .001
